# Adsorption of Congo red dye in water by orange peel biochar modified with CTAB

**DOI:** 10.1039/d3ra01444d

**Published:** 2023-04-21

**Authors:** Zhongxin Hua, Yaping Pan, Qiankun Hong

**Affiliations:** a Zhejiang Zhongda Engineering Costing Firm Co., Ltd Hangzhou 310012 China; b Zhejiang Tongji Vocational College of Science and Technology Hangzhou 311231 China hongqk0918@163.com

## Abstract

In order to improve the adsorption effect of biochar on Congo red dye, this study used hexadecyl trimethyl ammonium bromide (CTAB) to organically modify orange peel biochar (OBC) to produce CTAB-modified orange peel biochar (NOBC), and the biochar before and after modification was analyzed by SEM-EDS, FTIR and BET. The adsorption performance of NOBC on Congo red dye was investigated and the adsorption mechanism was studied. The results showed that the adsorption amount was influenced by the initial concentration, adsorption time and solution pH. NOBC adsorbed 50 mg L^−1^ CR with an equilibrium time of 60 min and an equilibrium amount of 290.1 mg g^−1^, while the adsorption equilibrium time of OBC was 210 min and an equilibrium amount of 155.2 mg g^−1^, the adsorption of CR by NOBC was above 210 mg g^−1^ at pH 2 to 11, NOBC can be recycled three times. The experimental results showed that the adsorption data of CR on NOBC were consistent with the Langmuir isothermal adsorption model and the Pseudo-second-order model, and the mechanism of CR adsorption on NOBC mainly included electrostatic attraction and surface adsorption. In conclusion, NOBC is a promising material for dye wastewater adsorption.

## Abstract 2

This study used hexadecyl trimethyl ammonium bromide (CTAB) to organically modify orange peel biochar (OBC) to produce CTAB-modified orange peel biochar (NOBC), the adsorption performance of NOBC on Congo red dye in water and the influencing factors were investigated.

## Graphical abstract

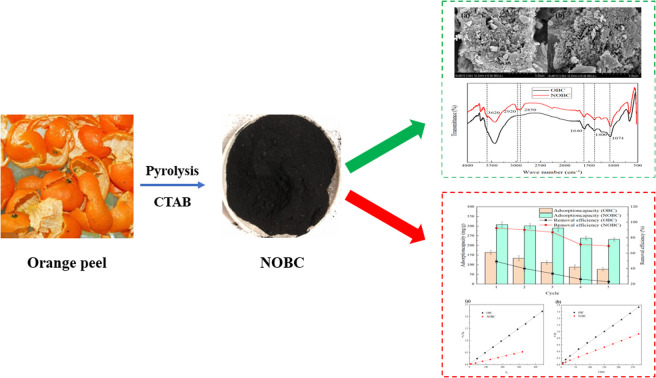

## 1 Introduction

Dyes are widely used in the leather, textile, pharmaceutical, food and dyeing industries, and as a result, the discharge of industrial wastewater contains high levels of dye pollutants, making it one of the major sources of pollution in China's water environment.^1,2^ Among them, Congo red (CR) is an anionic dye that is insoluble in acids and bases and is difficult to remove once it enters nature, not only inhibiting the growth and development of plants and animals, but also affecting human digestion, blood and cardiovascular systems to varying degrees.^3,4^ At present, the main methods for treating dye wastewater are: membrane separation,^5^ biodegradation,^6^ photocatalytic degradation,^7^ chemical oxidation^8,9^ and adsorption.^10,11^ Among them, adsorption is widely used due to the advantages of a flexible process, simple operation and high efficiency,^4^ but for any adsorption method to treat wastewater, its adsorption efficiency depends on the selection of a suitable adsorbent,^12,13^ therefore, the development of efficient and inexpensive adsorbent materials is key to promoting its practical application.

Biochar is a porous carbon material formed by high-temperature pyrolysis of biomass under anaerobic or anoxic conditions, not only having a high specific surface area, porosity and abundant functional groups, but is also prepared from a wide range of sources and simple preparation processes, and has been successfully used for the treatment of organic and inorganic polluted water bodies, creating great possibilities for the resource utilization of waste biomass.^13–15^ In order to improve the adsorption performance of biochar, it needs to be modified, mainly by changing its specific surface area and pore structure.^16^ This is because high adsorption efficiency is associated with a high specific surface area and large pore capacity. In addition, a suitable pore structure is important for good adsorption performance and can greatly accelerate the removal rate.^17^ Physical, biological and especially chemical modifications are effective methods to improve the adsorption capacity of adsorbents.^18^ CTAB, an inexpensive and widely used cationic surfactant, not only improves the layer spacing and specific surface area of the adsorbent material, but also enhances the adsorption of the anionic dye Congo red through electrostatic effects.^19^ Therefore, CTAB-modified biochar is an effective way to improve the adsorption capacity of biomass char. Therefore, CTAB-modified biochar is an effective method to improve the adsorption capacity and selectivity of anionic dyes.

In this study, the common waste orange peel was used as the biochar raw material and modified with CTAB to obtain modified orange peel biochar. The adsorption performance of modified orange peel biochar on Congo red dye in water and the influencing factors were investigated. The study provides a new way of thinking for the preparation of biochar and its utilisation, and also lays a solid foundation for the resourceful utilisation of waste and the pollution treatment of dyes in water.

## 2 Materials and methods

### 2.1 Materials

Orange peel was purchased from Puyang Luyuan Renewable Energy Technology Co., Ltd. CTAB, NaOH, H_3_PO_4_ and CR are analytically pure.

### 2.2 Methods

#### 2.2.1 Preparation of OBC and NOBC

The collected orange peels were washed and dried, then put into a high speed grinder and broken into small pieces of about 3–4 cm, then soaked in 2% NaOH solution for 24 h. After 24 h, the peels were washed with deionized water until neutral, put into an oven at 80 °C until completely dried, then activated for 24 h at room temperature by adding 85% phosphoric acid solution at a solid to liquid ratio of 1 : 4. The activated orange peel was put into a crucible and charred in a muffle furnace at 500 °C for 2 h. The powder was cooled to room temperature and passed through a 200 mesh sieve to obtain black OBC powder.

Dissolve 0.64 g CTAB powder in 200 mL ultrapure water, add 10 g OBC, stir for 2 h at 60 °C, centrifuge and discard the supernatant, then wash the pellet repeatedly with ultrapure water.

#### 2.2.2 Characterization of OBC and NOBC

The specific surface area and pore characteristics were determined by V-sorb 2800P pore size analyzer (Gold APP, China), where the specific surface area was calculated based on the multi-point BET (Brunauer–Emmett–Teller) adsorption isotherm. The specific surface area was calculated according to the multipoint BET (Brunauer–Emmett–Teller) adsorption isotherm, the mesopores and macropores according to the Barrett–Joyner–Halenda method and the micropores according to the Saito–Foley method.

#### 2.2.3 Adsorption experiments

Weigh 1.0 g of CR, dry at 105 °C for 2 h, add deionised water to a volume of 1000 mL and prepare a CR standard stock solution of 1000 mg L^−1^. The CR staining solution used in the experiment was obtained by diluting the above standard stock solution. Shake at 25 °C at 130 rpm.

##### 2.2.3.1 Effect of adsorption time on adsorption

0.4 g L^−1^ of OBC and NOBC were added to a series of conical flasks containing 50 mL and 50 mg L^−1^ of CR staining solution, respectively, and shaken in a constant temperature water bath shaker. The conical flasks were removed at different time intervals from 0 to 300 min and the absorbance of CR in the supernatant was measured at a wavelength of 488 nm to calculate the removal rate of the CR and to analyse the effect of adsorption time on the adsorption effect of the dye.

1 *q* = (*c*_0_ − *c*) V m^−1^

2 *η* = (*c*_0_ − *c*)/*c*_0_ × 100%

where *q* is the adsorption capacity of OBC and NOBC, mg g; *c*_0_ is the concentration of CR solution before adsorption, mg L^−1^; *c* is the concentration of remaining CR solution after adsorption, mg L^−1^; *V* is the volume of CR solution, L; *m* is the mass of OBC and NOBC, g.

##### 2.2.3.2 Effect of initial pH on adsorption

The pH of the CR dye solution was adjusted with 0.5 mol L^−1^ HCl and 1 mol L^−1^ NaOH. 0.4 g L^−1^ of adsorbent was added to 50 mg L^−1^ of CR dye solution and shaken to reach equilibrium, and the supernatant was centrifuged to measure the absorbance of the dye and calculate its removal rate to study the effect of the initial pH of the dye solution on the adsorption.

##### 2.2.3.3 Effect of adsorbent dosage on adsorption

A certain amount of biochar was added to the CR solution and the adsorbent dosage was set at 0.2–1.2 g L^−1^, set at 25 °C and shaken for 36 h. The removal rate and adsorption capacity were calculated.

#### 2.2.4 Recyclability experiment

0.06 g of OBC and NOBC were added into 150 mL and 100 mg L^−1^ of CR solution, respectively, and after the adsorption saturation, the samples were desorbed in H_2_O_2_ solution with a concentration of 0.003 mmol L^−1^ for 1 h. The samples were washed with deionized water several times and then centrifuged, and then dried in a constant temperature drying oven at 60 °C for 6 h. The dried samples were repeated four times according to the above procedure for the recyclability experiment.

## 3 Results and discussion

### 3.1 Characterization analysis of OBC and NOBC

**Fig. 1:**
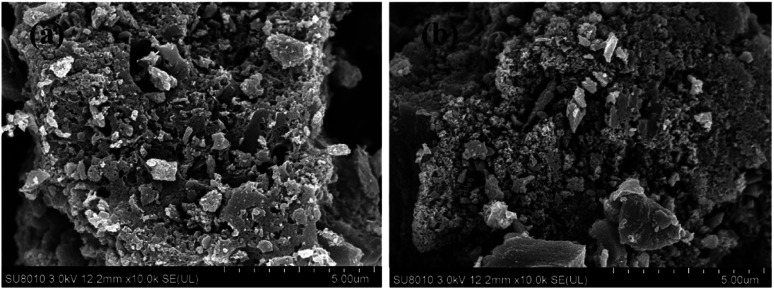
Orange peel biochar before and after modification, (a) before modification; (b) after modification.

Fig. 1 shows the scanning electron microscopy of OBC before and after modification by CTAB. There were more blocky structures on the surface of both biochar, with large spacing between each structure and uneven folds and rough surfaces, which provided active sites for the adsorption of Congo red by biochar and made the biochar have better adsorption performance.^19^ After modification by CTAB, the surface of NOBC showed no obvious changes, indicating that CTAB had no obvious effect on the morphology of OBC, which mainly improves the adsorption of CR by changing the surface polarity of the biochar.^20,21^

**Fig. 2:**
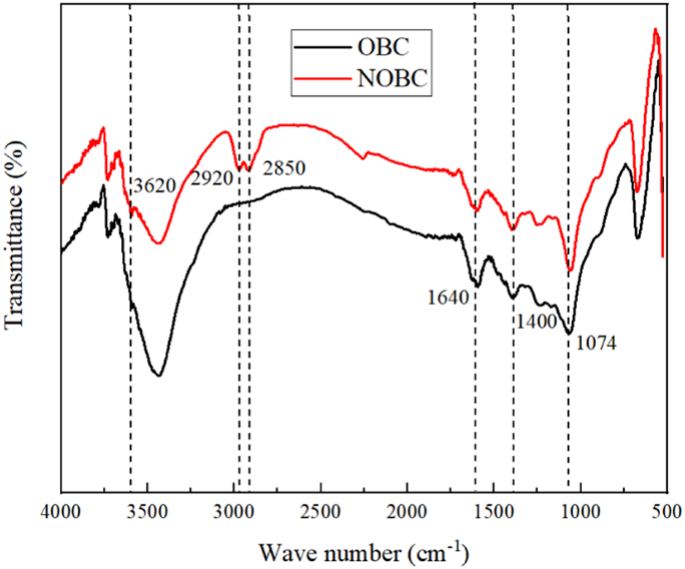
FT-IR spectra of OBC and NOBC.

As can be seen in Fig. 2, the FTIR spectra of OBC and CTAB/OBC show that the peaks of OBC and CTAB/OBC are similar in shape, indicating that the basic skeleton of OBC is not destroyed during the modification of OBC by CTAB. The peaks at 3620, 1640 and 1040 cm^−1^ correspond to the hydroxyl –OH stretching vibration absorption peaks of the structural water of the biomass carbon, the bending vibration absorption peaks of the hydroxyl –OH of the interlayer adsorbed water and the –COOH stretching vibration absorption peaks in the lattice, respectively. The peaks at 2920 and 2850 cm^−1^ correspond to the stretching vibrational peaks of methyl –CH_3_ and –CH_2_, respectively. Thus, OBC successfully combined with CTAB in the modification process to form CTAB/OBC.

Since the biochar generated through the pyrolysis process produces many new micropores,^22^ the biomass char before and after the modification has a large specific surface area, indicating that the biochar before and after the modification has a strong adsorption capacity. As shown in Table 1, the reduction in the specific surface area of NOBC compared to OBC was from 697.05 to 618.44 m^2^ g^−1^. The reduction in the specific surface area of NOBC may be due to the adhesion of the CTAB modifier to the surface of the BC, resulting in the blockage of the pores of the biochar.^23,24^

**Table 1:** Specific surface area, pore volume, and pore structure of OBC and NOBC

Sample	BET surface area (m^2^ g^−1^)	Micropore surface area (m^2^ g^−1^)	Pore volume (cm^3^ g^−1^)	Pore diameter (μm)
OBC	697.05	477.42	0.28	4.26
NOBC	618.44	453.18	0.27	7.28

**Fig. 3:**
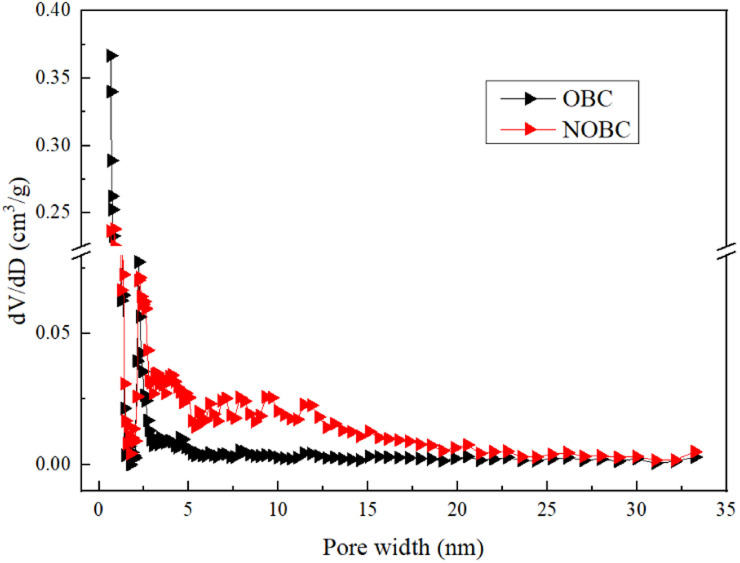
Pore size distribution of OBC and NOBC.

To further obtain the pore size range of the material, the pore size distribution obtained from the DFT model calculation is shown in Fig. 3, it can be seen that the pore size of OBC is mainly concentrated in the range of 0.5–3 nm, while the pore size of NOBC is concentrated in the range of 0.1–15 nm, indicating that the CTAB modification is able to improve the pore structure of the OBC surface, making it more abundant in micro- and mesopores.

### 3.2 Adsorption influencing factors

#### 3.2.1 Adsorbent dosing

**Fig. 4:**
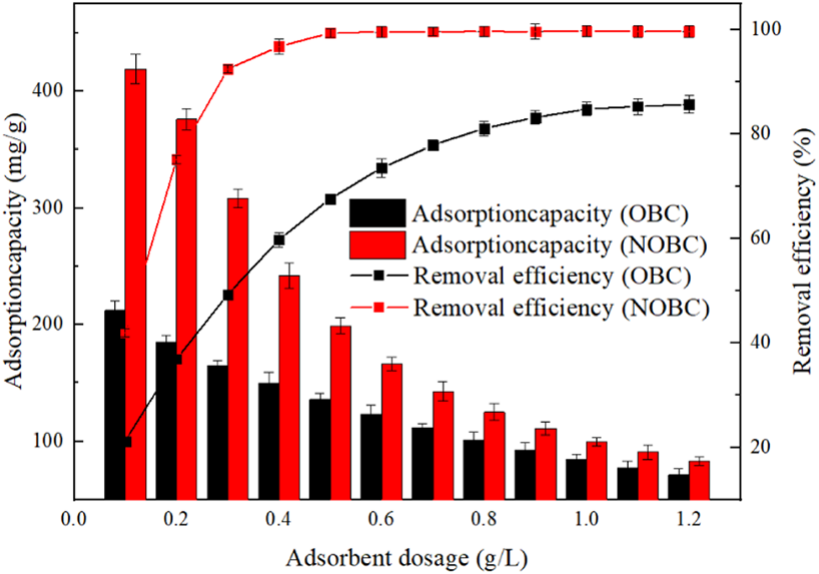
Effect of sorbent dosing on the sorption CR of OBC and NOBC.

When different masses of OBC and NOBC were added to 100 mg L^−1^ of Congo red solution, the effect of OBC and NOBC dosage on the adsorption of Congo red can be seen as shown in Fig. 4. It can be found that the adsorption efficiency of Congo red increased with the increase of OBC and NOBC dosage, while the unit adsorption amount showed different degrees of decrease. When the dosage was increased from 0.1 g L^−1^ to 1.2 g L^−1^, the removal rates of Congo red by OBC and NOBC increased from 21.2% and 41.9% to 85.7% and 99.75%, respectively, and the unit adsorption amounts decreased from 212 mg g^−1^ and 419 mg g^−1^ to 71.4 mg g^−1^ and 83.12 mg g^−1^, respectively, which was mainly due to the fact that under the condition of constant Congo red concentration This was mainly due to the increase in surface area and effective adsorption sites as the adsorbent dosage increased, thus increasing the removal rate under constant Congo red concentration.^25^ The adsorption effect of NOBC on Congo red was much higher than that of OBC, probably due to the positive charge of CTAB on the surface of NOBC, which greatly facilitated the adsorption of anionic dyes through electrostatic attraction.^26^

#### 3.2.2 Adsorption time

**Fig. 5:**
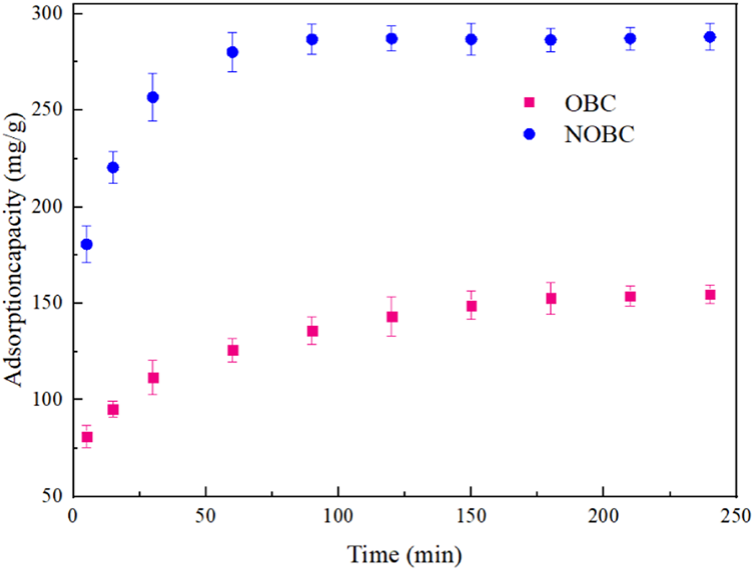
Effect of adsorption time on the adsorption CR of OBC and NOBC.

The adsorption at different contact times and the subsequent time to reach adsorption equilibrium is an important parameter of the adsorption process.^27^ The effect of contact time on the adsorption effect of Congo red is shown in Fig. 5. In the early stage of adsorption, both OBC and NOBC adsorbed Congo red at a faster rate, which was mainly due to the sufficient active sites on the surface of the adsorbent at the beginning. As time progressed, the adsorbent gradually filled the active sites of the adsorbent, adsorption became increasingly difficult and the rising trend of adsorption slowed down, eventually reaching adsorption equilibrium at 210 min and 60 min for OBC and NOBC respectively, with adsorption equilibrium amounts of 155.2 mg g^−1^ and 290.1 mg g^−1^ respectively. The adsorption rate and efficiency of NOBC was higher than that of OBC. This was mainly due to the fact that CTAB could greatly enhance the electrostatic interaction between NOBC and Congo red, which in turn increased the adsorption rate and amount of NOBC on Congo red.

#### 3.2.3 pH

**Fig. 6:**
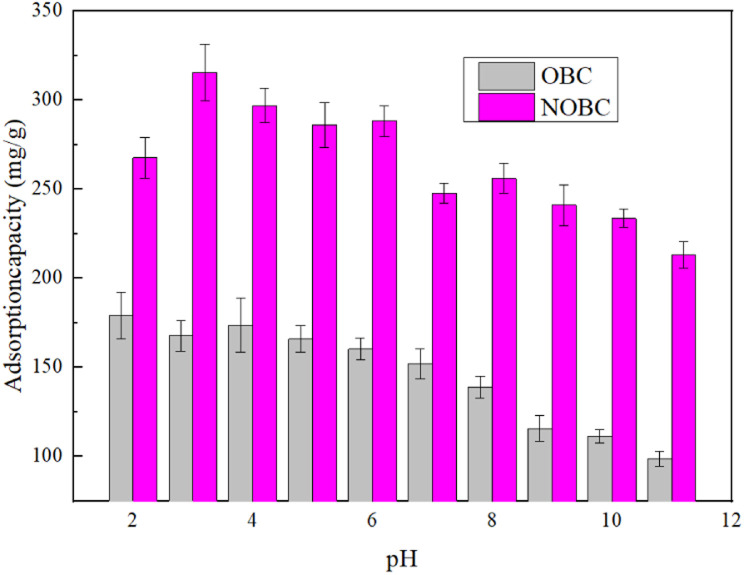
Effect of pH on CR adsorption by OBC and NOBC.

Fig. 6 shows the effect of pH on the adsorption capacity of OBC and NOBC. The results show that the adsorption capacity of both OBC and NOBC has a significant effect. For OBC, the adsorption capacity showed a gradual decrease as the pH increased, with the adsorption capacity of OBC being only 98.67 mg g^−1^ at pH 11.0. For NOBC, the adsorption capacity of NOBC increased to 315.63 mg g^−1^ when the pH increased from 2.0 to 3.0. When the pH was greater than 3.0, the adsorption capacity of NOBC for Congo red gradually declined.

**Fig. 7:**
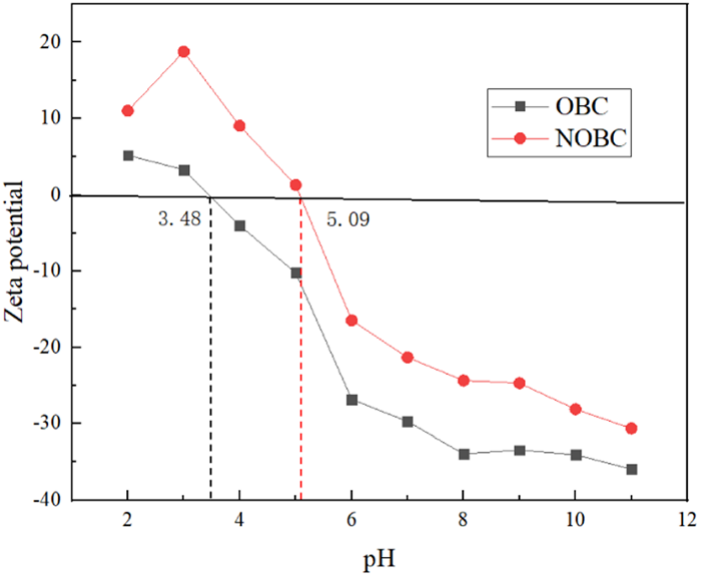
Zeta potential of OBC and NOBC.

To further investigate the effect of pH on the surface charges of OBC and NOBC, we studied the potentials of OBC and NOBC at different pH conditions. Fig. 7 shows that the zero potential points of OBC and NOBC were 3.48 and 5.09 respectively. When the pH was less than 3.48, the OBC surface was positively charged, which contributed to the adsorption of Congo red. When the pH was greater than 3.48, the negative charge on the OBC surface increased, and the electrostatic attraction with Congo red gradually decreased, so the adsorption capacity decreased. When the pH value is less than 5.09, the surface of NOBC is positively charged, and when pH = 3.0 the positive charge is the largest, at this time the electrostatic attraction between NOBC and Congo red is the largest, and the adsorption effect is the best. When the pH value is greater than 5.09, as the pH value increases, the electro-negativity of the NOBC surface gradually increases, and the adsorption effect decreases.^28^

### 3.3 Adsorption isotherms and adsorption kinetic models

#### 3.3.1 Adsorption isotherms

**Fig. 8:**
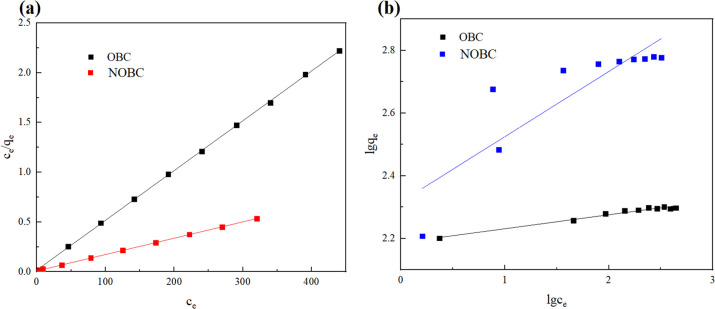
Adsorption isotherm of OBC and NOBC adsorption CR (a: Langmuir adsorption isotherm, b: Freundlich adsorption isotherm).

Adsorption isotherms can elucidate the interaction between the adsorbed material and the adsorbent and contribute to the understanding of the adsorption process.^29^ The Langmuir adsorption isotherm model and the Freundlich adsorption isotherm model were used to analyse the adsorption equilibrium of OBC and NOBC, as shown in Fig. 8 and Table 2.

**Table 2:** Isotherm parameters of CR adsorption on OBC and NOBC

Sample	Langmuir adsorption isotherm equation			Freundlich adsorption isotherm equation		
	*q*_m_ (mg g^−1^)	*K*_L_ (L mg^−1^)	*R* ^2^	*K*_F_	*n*	*R* ^2^
OBC	200	0.308	0.999	153.78	22.52	0.972
NOBC	609.8	0.196	0.999	207.45	4.81	0.739

When fitted with the Langmuir adsorption isotherm model, the correlation coefficients for CR adsorption by OBC and NOBC were both 0.999. The Langmuir adsorption capacities of OBC and NOBC for CR were 200 and 609.8 mg g^−1^, respectively, which were similar to the actual measured adsorption values, demonstrating that the adsorption process was consistent with the Langmuir model and that CR adsorption on the two biochars occurred the *K*_L_ values for CR adsorption on NOBC were significantly greater than those for OBC, indicating that NOBC has a stronger capacity for dye adsorption,^30^ Freundlich's adsorption index, reflects the magnitude of the interaction between the adsorbent and the adsorbate, and when 1/*n* is less than 1, the adsorption reaction is easy to proceed, and the adsorption of CR on either OBC or NOBC are readily carried out.^31^

#### 3.3.2 Adsorption kinetic model

**Fig. 9:**
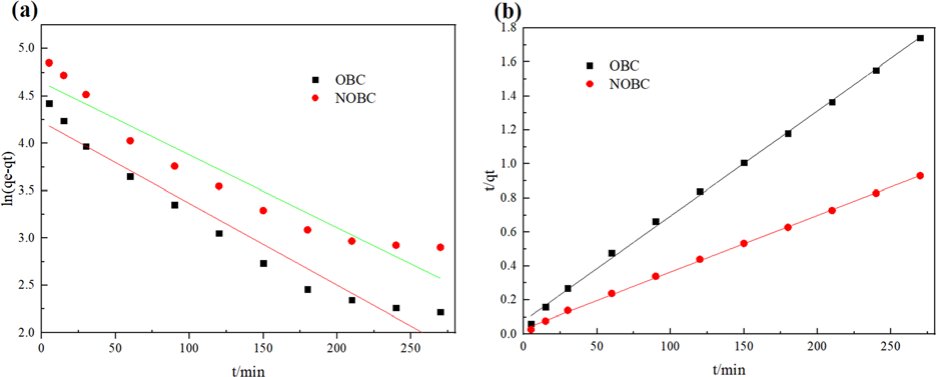
Adsorption kinetic curve of OBC and NOBC adsorption CR (a: Pseudo-first-order model, b: Pseudo-second-order model).

The kinetic correlation coefficients in Fig. 9 and Table 3 and the comparison between qe and qm indicate that the adsorption of CR by OBC and NOBC is more in line with the quasi-secondary kinetic model.^32^ A comparison of the *K*_2_ values shows that NOBC adsorbs CR at a faster rate than OBC [40]. The results of the fit of the intraparticle diffusion model indicate that the adsorption of CR by OBC and NOBC starts with a rapid adsorption phase, then the adsorption rate slows down and finally the adsorption reaches equilibrium and stability, and, 0.1 < *C* < 1, indicating that there is intraparticle diffusion in the adsorption process.^33,34^

**Table 3:** Kinetics parameters of CR adsorption on OBC and NOBC

Sampe	OBC	NOBC
*q*_e,exp_ (mg g^−1^)	164.33	308.33

Pseudo-first-order model		
*q*_e,calc_ (mg g^−1^)	68.63	63.43
*K*_1_ (min^−1^)	0.0086	0.0077
*R*_1_^2^	0.9450	0.5780

Pseudo-second-order model		
*q*_e,calc_ (mg g^−1^)	161.81	292.40
*K*_2_ (g mg^−1^ min^−1^)	0.00050	0.00037
*R*_2_^2^	0.998	0.999

Intraparticle diffusion model		
*C*	0.7937	0.9233
*K*_p_	5.2298	6.1633
*R*_3_^2^	0.9290	0.6581

### 3.4 Recycling

**Fig. 10:**
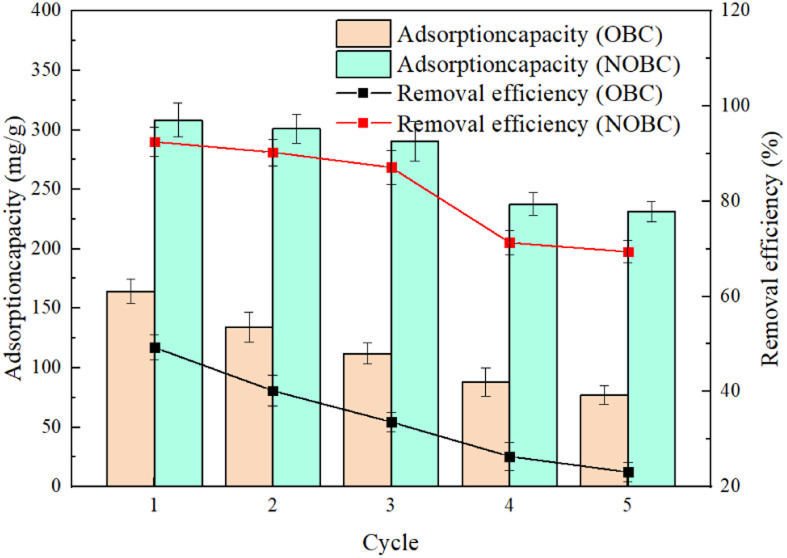
CR adsorption recycles on OBC and NOBC.

The reusability of the adsorbent is an important indicator to evaluate whether the adsorbent has practical application value. As shown in the Fig. 10, the unit adsorption amount and adsorption efficiency of NOBC and OBC both decreased to different degrees after 5 adsorption desorptions, which may be due to some adsorption sites in NOBC and OBC were not completely desorbed. It is worth mentioning that the unit adsorption amount of OBC decreased from 164.3 mg g^−1^ to 77 mg g^−1^ after 5 adsorption desorptions, the adsorption removal rate decreased from 49.3% to 23.1%, and the unit adsorption amount of NOBC decreased from 308.3 mg g^−1^ to 231.3 mg g^−1^ after 5 adsorption desorptions. Further, there is a possibility that the reduction in adsorption capacity after the third cycle in the recycling section could be explained by the gradual desorption of the CTAB molecules from the surface. This shows that NOBC has more advantages than OBC in terms of reusability, and NOBC has good stability and reusability in 3 cycles of recycling. NOBC can be reused 3 times.

## 4 Conclusions

In this study, CTAB was used to organically modify OBC to obtain NOBC materials with good stability and good adsorption performance, and the main conclusions are as follows.

(1) CTAB was successfully loaded on the surface of OBC, forming a multi-stage pore adsorption material, with the specific surface area reduced from 697.05 to 618.44 m^2^ g^−1^ and the average pore size increased from 4.26 to 7.28 nm.

(2) Compared with OBC, the adsorption capacity of NOBC for CR was significantly enhanced, and the corresponding Langmuir adsorption capacity increased from 200 mg g^−1^ to 609.8 mg g; the pH value of the dye solution had a more obvious effect on the biomass carbon before and after modification, and the adsorption capacity tended to decrease as the pH value increased; after five times of reuse experiments, the removal rate of CR by NOBC was still maintained at over 70%. The stability and recycling ability of the material were significantly better than those of OBC.

(3) The adsorption of NOBC on CR dyeing solution was in accordance with the Langmuir model, and the corresponding Langmuir adsorption capacity was 609.8 mg g^−1^. The adsorption process was in accordance with the quasi-secondary kinetic model, and the main adsorption mechanisms included electrostatic attraction and surface adsorption.

## Author contributions

Z. X. Hua: conceptualization, funding acquisition, supervision conceptualization, investigation, methodology, writing – original draft, writing – review & editing. Y. P. Pan: methodology, writing – original draft, writing – review & editing. Q. K. Hong: investigation, writing – original draft, supervision, funding acquisition. All authors have read and agreed to the published version of the manuscript.

## Conflicts of interest

The authors declare no potential conflict of interest.

## Supplementary Material

## References

[cit1] Ortiz-Monsalve S., Dornelles J., Poll E. (2017). *et al.*, Biodecolourisation and biodegradation of leather dyes by a native isolate of Trametes villosa. Process Saf. Environ. Prot..

[cit2] Pathy A., Pokharel P., Chen X. L. (2023). *et al.*, Activation methods increase biochar's potential for heavy-metal adsorption and environmental remediation: A global meta-analysis. Sci. Total Environ..

[cit3] Gao L., Li Z. H., Yi W. M. (2021). *et al.*, Impacts of pyrolysis temperature on lead adsorption by cotton stalk-derived biochar and related mechanisms. J. Environ. Chem. Eng..

[cit4] Xiang W., Zhang X. Y., Luo J. P. (2022). *et al.*, Performance of lignin impregnated biochar on tetracycline hydrochloride adsorption: Governing factors and mechanisms. Environ. Res..

[cit5] Masoudian N., Rajabi M., Ghaedi M. (2019). Titanium oxide nanoparticles loaded onto activated carbon prepared from bio-waste watermelon rind for the efficient ultrasonic-assisted adsorption of congo red and phenol red dyes from wastewaters. Polyhedron.

[cit6] Dai L. C., Zhu W. K., He L. (2018). *et al.*, Calcium-rich biochar from crab shell: An unexpected super adsorbent for dye removal. Bioresour. Technol..

[cit7] Yang X. Y., Zhu W. F., Chen F. Y. (2023). *et al.*, Modified biochar prepared from Retinervus luffae fructus for dyes adsorption and aerobic sludge granulation. Chemosphere.

[cit8] Huang W. H., Wu R. M., Chang J. (2023). Manganese ferrite modified agricultural waste-derived biochars for copper ions adsorption. Bioresour. Technol..

[cit9] Ramírez G., Recio F. J., Herrasti P. (2016). *et al.*, Effect of RVC porosity on the performance of PbO2 composite coatings with titanate nanotubes for the electrochemical oxidation of azo dyes. Electrochim. Acta.

[cit10] Wong S., Tumari H. H., Ngadi N. (2019). Adsorption of anionic dyes on spent tea leaves modified with Polyethyleneimine (PEISTL). J. Cleaner Prod..

[cit11] Holkar C. R., Jadhav A. J., Pinjari D. V. (2016). A critical review on textile wastewater treatments: possible approaches. J. Environ. Manage..

[cit12] Du L. Q., Ahmad S., Liu L. N. (2023). *et al.*, A review of antibiotics and antibiotic resistance genes (ARGs) adsorption by biochar and modified biochar in water. Sci. Total Environ..

[cit13] Park J. H., Wang J. J., Meng Y. L. (2019). *et al.*, Adsorption/desorption behavior of cationic and anionic dyes by biochars prepared at normal and high pyrolysis temperatures. Colloids and Surfaces A: Physicochemical and Engineering Aspects.

[cit14] Dai Y. J., Zhang N. X., Xing C. M. (2019). *et al.*, The adsorption, regeneration and engineering applications of biochar for removal organic pollutants: A review. Chemosphere.

[cit15] Shakya A., Vithanage M., Agarwal T. (2022). Influence of pyrolysis temperature on biochar properties and Cr(VI) adsorption from water with groundnut shell biochars: Mechanistic approach. Environ. Res..

[cit16] Nguyen X. C., Nguyen T., Nguyen T. (2021). *et al.*, Sustainable carbonaceous biochar adsorbents derived from agro-wastes and invasive plants for cation dye adsorption from water. Chemosphere.

[cit17] Xiang W., Zhang X. Y., Chen at al. J. J. (2020). Biochar technology in wastewater treatment: a critical review. Chemosphere.

[cit18] Zhao Y. L., Zhang R. Y., Liu H. B. (2019). *et al.*, Green preparation of magnetic biochar for the effective accumulation of Pb(II): Performance and mechanism. Chem. Eng. J..

[cit19] Meneses L. P., Novaes S. D., Dezotti R. S. (2022). *et al.*, CTAB-modified carboxymethyl cellulose/bagasse cryogels for the efficient removal of bisphenol A, methylene blue and Cr(VI) ions: Batch and column adsorption studies. J. Hazard. Mater..

[cit20] Yang X. X., Li Y. M., Gao H. M. (2018). *et al*, One-step fabrication of chitosan-Fe(OH)_3_ beads for efficient adsorption of anionic dyes. Int. J. Biol. Macromol..

[cit21] Shi Y., Du J. D., Zhao T. M. (2023). *et al*, Removal of nanoplastics from aqueous solution by aggregation using reusable magnetic biochar modified with cetyltrimethylammonium bromide. Environ. Pollut..

[cit22] Zhu J. Q., Li J. Y., Li Y. Y. (2019). *et al.*, Adsorption of phosphate and photodegradation of cationic dyes with BiOI in phosphate-cationic dye binary system. Sep. Purif. Technol..

[cit23] Ghuge S. P., Saroha A. K. (2018). Catalytic ozonation of dye industry effluent using mesoporous bimetallic Ru-Cu/SBA-15 catalyst. Process Saf. Environ. Prot..

[cit24] Rath P. P., Behera S. S., Priyadarshini B. (2019). *et al.*, Influence of Mg doping on ZnO NPs for enhanced adsorption activity of Congo Red dye. Appl. Surf. Sci..

[cit25] Wang Y., Yang Q. X., Chen J. C. (2020). *et al.*, Adsorption behavior of Cr(VI) by magnetically modified Enteromorpha prolifera based biochar and the toxicity analysis. J. Hazard. Mater..

[cit26] Ortiz-Monsalve S., Dornelles J., Poll E. (2017). *et al.*, Biodecolourisation and biodegradation of leather dyes by a native isolate of Trametes villosa. Process Saf. Environ. Prot..

[cit27] Prajapati A. K., Mondal M. K. (2022). Green synthesis of Fe3O4-onion peel biochar nanocomposites for adsorption of Cr(VI), methylene blue and congo red dye from aqueous solutions. J. Mol. Liq..

[cit28] Lei C. S., Zhu X. F., Zhu B. C. (2017). *et al.*, Superb adsorption capacity of hierarchical calcined Ni/Mg/Al layered double hydroxides for Congo red and Cr(VI) ions. J. Hazard. Mater..

[cit29] Rubangakene N. O., Elwardany A., Fujii M. (2023). *et al.*, Biosorption of Congo Red dye from aqueous solutions using pristine biochar and ZnO biochar from green pea peels. Chem. Eng. Res. Des..

[cit30] Sewu D. D., Boakye P., Jung H. (2017). *et al.*, Synergistic dye adsorption by biochar from co-pyrolysis of spent mushroom substrate and Saccharina japonica. Bioresour. Technol..

[cit31] Sun Y. C., Wang T. T., Han C. H. (2023). *et al.*, One-step preparation of lignin-based magnetic biochar as bifunctional material for the efficient removal of Cr(VI) and Congo red: Performance and practical application. Bioresour. Technol..

[cit32] Yao X. X., Ji L. L., Guo J. (2020). *et al.*, Magnetic activated biochar nanocomposites derived from wakame and its application in methylene blue adsorption. Bioresour. Technol..

[cit33] Ma M. J., Ying H. J., Cao F. F. (2020). *et al.*, Adsorption of congo red on mesoporous activated carbon prepared by CO_2_ physical activation. Chin. J. Chem. Eng..

[cit34] Sewu D. D., Boakye P., Woo S. H. (2017). Highly efficient adsorption of cationic dye by biochar produced with Korean cabbage waste. Bioresour. Technol..

